# Effect of Psychological Intervention on Pelvic Floor Function and Psychological Outcomes After Hysterectomy

**DOI:** 10.3389/fmed.2022.878815

**Published:** 2022-04-25

**Authors:** Meilian Xie, Xin Huang, Shan Zhao, Yingtong Chen, Xiuqun Zeng

**Affiliations:** Department of Gynecology, Guangdong Provincial People's Hospital, Guangdong Academy of Medical Sciences, Guangzhou, China

**Keywords:** pelvic floor function, depression, anxiety, hysterectomy, psychological intervention

## Abstract

**Background:**

Hysterectomy is one of the most frequently performed operations worldwide. However, postoperative complications and body image changes may induce psychological distress after hysterectomy. The study aimed to evaluate the effect of psychological intervention on pelvic floor function and psychological outcomes following hysterectomy among patients with benign indications.

**Methods:**

Ninety-nine patients underwent hysterectomy were randomly divided into intervention group (*n* = 50) and control group (*n* = 49). Patients in the control group received routine postoperative nursing care, while extra psychological intervention was provided to patients in the intervention group, including psychological support, regular lectures and family support. After 6 months, patient's psychological statuses were assessed by Generalized Anxiety Disorder scale (GAD-7) and Patient Health Questionnaire-9 (PHQ-9). The pelvic floor function of patients was evaluated using Pelvic Floor Impact Questionnaire (PFIQ-7) and Pelvic Organ Prolapse/Urinary Incontinence Sexual Questionnaire (PISQ-12). Furthermore, the incidence of postoperative complications, including uracratia, pelvic organ prolapses, sexual dysfunction and chronic pelvic pain, was evaluated.

**Results:**

After 6-month intervention, the GAD-7 and PHQ-9 scores were significantly decreased in the intervention group (*p* < 0.001 and *p* = 0.018 respectively). Both scored were significantly lower than that in the control group (*p* < 0.001 and *p* < 0.001). Compared with control group, the incidence of uracratia, pelvic organ prolapse, sexual dysfunction and chronic pelvic pain for intervention group was significantly lower (*p* = 0.003, *p* = 0.027, *p* = 0.001, *p* = 0.002 respectively) and the pelvic floor muscle strength was significantly stronger (*p* = 0.001). Besides, the postoperative Urinary Incontinence Impact Questionnaire (UIQ-7), Pelvic Organ Prolapse Impact Questionnaire (POPIQ-7), and Colorectal-Anal Impact Questionnaire (CRAIQ-7) scores were significantly lower (*p* = 0.025, *p* = 0.04, *p* < 0.001) and PISQ-12 score was significantly higher in intervention group (*p* < 0.001).

**Conclusion:**

Psychological intervention could effectively improve the psychological condition of patients with hysterectomy, which may facilitate patients' postoperative recovery in pelvic floor function. These findings emphasized the necessity of psychological intervention in routine postoperative nursing care.

## Introduction

Hysterectomy is a common gynecological procedure for various benign indications, such as hysteromyoma, endometrioma, deep infiltrating endometriosis, dysmenorrhea and dysfunctional uterine bleeding (1–5). Although hysterectomy rates have declined globally due to the application of conservative treatment, it still remains the second most common therapeutic modality (6, 7). There were more than 400,000 patients underwent hysterectomies annually in European Union (8) and about 33% of women in United States had hysterectomies before 60 years old (9). However, like any other surgery, there are a number of postoperative complications after hysterectomy, ranging from infection to anatomical injuries (10, 11). One of the most common long-term complications is pelvic organ prolapse (POP), with an incidence varying from 2~43% (12, 13). POP adversely affects the patient's personal, social and sexual activities. The most frequent symptoms reported included dyspareunia and sexual desire decreased (14, 15), which seriously reduce the quality of life and bring great psychological pressure of women (16). A cross-sectional study of 177 participants showed that one-third of women with POP had depressive symptoms (17). Besides, growing evidence indicated that women with hysterectomy suffered both physical and psychological injury which usually overlapped and interacted with each other (18). Therefore, it is necessary to investigate the psychological outcome of patients with hysterectomy throughout therapeutic process.

The psychological outcome of patients has long been the major issue of concern from both professional and public views. Many studies have shown that psychological interventions could improve the health status, physical role and emotional functions of cancer patients (19–21). A recent meta-analysis of 23 controlled trials, including 2,965 patients, reported that psychological interventions revealed moderate strength effects on quality of life (QOL) among cancer patients and survivors (22). Hence, psychological interventions may help to reduce psychological distress and increase QOL in patients with hysterectomy. Unfortunately, limited study has been devoted to explore this area. Thus, it deserves further investigation. The aim of present study was to detect the efficacy of psychological intervention on patient's pelvic floor function and psychological outcomes following hysterectomy.

## Materials and Methods

### Patients

**Figure 1:**
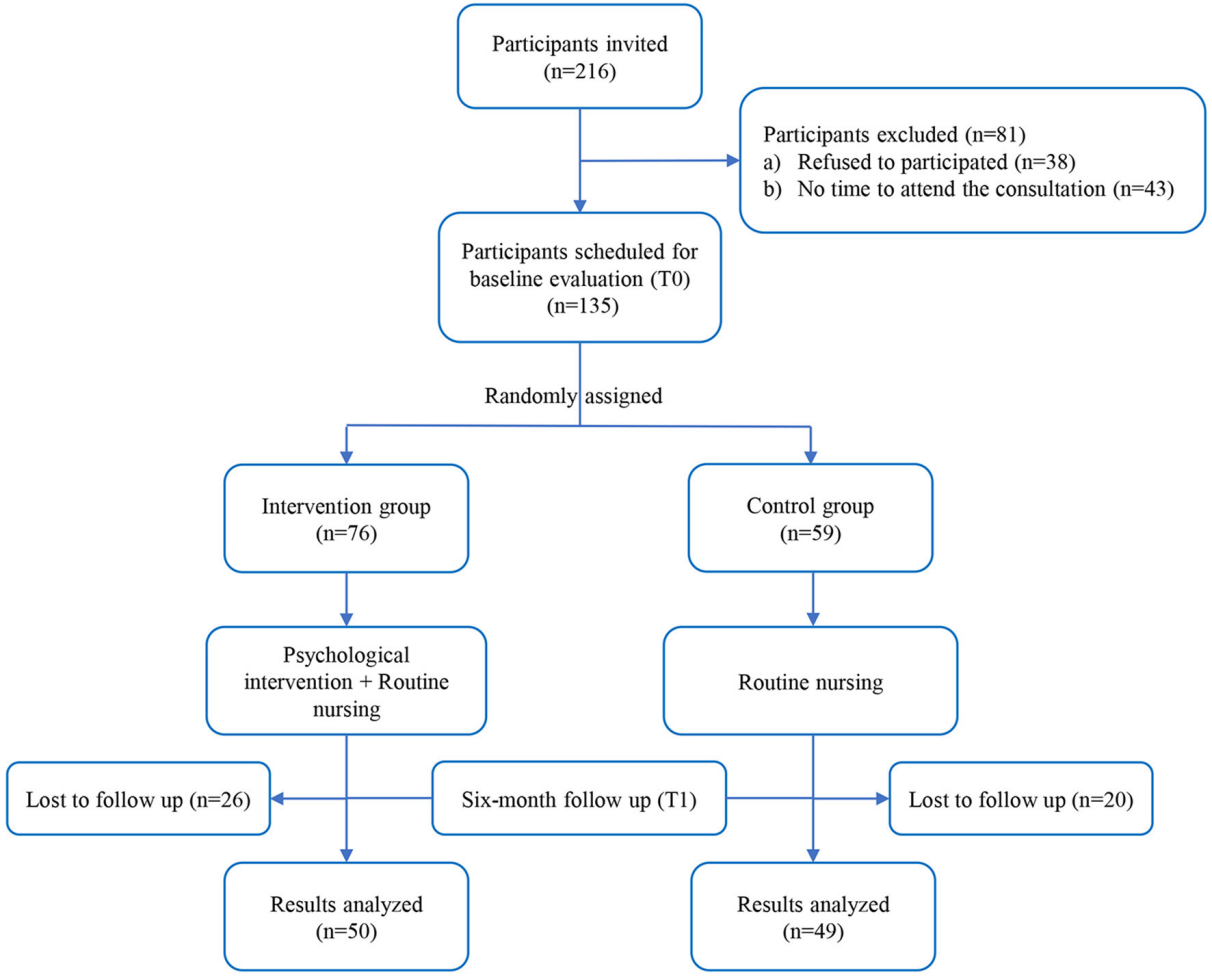
Flow diagram of the study, included and excluded.

Figure 1 showed the processes by which the study participants were selected. We finally included 99 patients underwent hysterectomy at Guangdong Provincial People's Hospital from May 2020 to May 2021. All participants underwent laparoscopic hysterectomy because of benign indications. Any subject with psychiatric disorders or cognitive illnesses before hysterectomy was excluded. The pelvic floor function of patients was evaluated before hysterectomy according to Pelvic Organ Prolapse Quantitation (POP-Q) (23). Any subject with pelvic floor dysfunction or pelvic floor deformation was also excluded from the study. A written informed consent was obtained from each patient prior to participating in this study. This study was approved by the Ethics Committee of Guangdong Provincial People's Hospital and was performed in accordance with the Code of Ethics of World Medical Association (Declaration of Helsinki).

### Procedures

All study participants received the initial evaluation before hysterectomy. The general information was investigated by questionnaires including marital status, educational level, occupation, economic conditions and so on. The initial psychological states of patients were evaluated, which were identified as baseline evaluation values (T0). After the initial evaluation, the eligible participants were randomly assigned into psychological intervention group or control group.

The patients in control group received regular nursing. After the hysterectomy was completed, routine health education was conducted to them.

The patients assigned to psychological intervention group were matched to one-to-one professional guidance by an independent investigator. In addition to conventional nursing, the close communication between investigators and patients was established and performed every 2 weeks though an instant messaging app. This helped to provide psychological support and ensure the participants were comfortable at all times. Some methods for reducing stress and controlling emotions were introduced to patients, such as listening to music, doing exercise, traveling, and cognitive restructuring and so on. If the professional psychological care was necessary, a psychiatrist or psychologist would be provided. In addition, the lectures for lifestyle behavior recommendation were held periodically. Investigators would regularly urge patients to exercise pelvic floor muscles. Family support was also a part of psychological intervention. Postoperative nursing knowledge was explained to patient's family, especially her husband. Investigators would guide them on how to concern, support and help patients after hysterectomy.

Six months after hysterectomy, all participants in both psychological intervention group and control group underwent the final assessments (T1), including patient's psychological status, pelvic floor function and the incidence of postoperative complications.

This study was compliant with the Strengthening the Reporting of Observational Studies in epidemiology (STROBE) Statement: guideline for cohort study (Supplementary File 1).

### Observations/Assessment Methods

The psychological status of patients was evaluated using GAD-7 and PHQ-9. The GAD-7 is a valid and efficient tool for screening generalized anxiety disorder (24). The severity of anxiety for the last 2 weeks is assessed based on the 7 core symptoms. The response options for each item are “not at all,” “several days,” “more than half the days,” and “nearly every day,” which scored as 0, 1, 2, and 3, respectively. The overall score ranges from 0 to 21 and higher scores indicate more severe symptoms of anxiety. PHQ-9 is a 9-item self-administered instrument designed to detect depression and assess the severity of depression symptoms (25). Similar to GAD-7, the item scores range from 0 (not at all) to 3 (nearly every day), resulting in a sum score range from 0 to 27.

The pelvic floor function was assessed by the Patient Questionnaire Form, Oxford Grading Scale, Pelvic Floor Impact Questionnaire (PFIQ-7) and Pelvic Organ Prolapse/Urinary Incontinence Sexual Questionnaire (PISQ-12). The Patient Questionnaire Form contained the questions to validate whether the patients had pelvic floor dysfunction or not, including uracratia, pelvic organ prolapse, sexual dysfunction and chronic pelvic pain. The pelvic floor muscle strength is assessed using the five-point Oxford Grading Scale (26). Patients are placed in the lithotomy position and instructed to squeeze their pelvic floor muscles as strongly as possible and then hold on. The muscle strength is assessed by digital examination of vagina and the test need to repeat 5 times. According to the pelvic floor muscle strength, the scales are divided into 5 degrees: grade 0 = no contraction; grade 1 = flicker; grade 2 = weak; grade 3 = moderate; grade 4 = good; and grade 5 = strong. The PFIQ-7 is used to assess how much the activities, relationships, or feelings of patients have been affected by their bladder, bowel, or vaginal symptoms in the past 3 months (27). It is composed of three scales with seven questions, which are Urinary Incontinence Impact Questionnaire (UIQ-7), Pelvic Organ Prolapse Impact Questionnaire (POPIQ-7), and Colorectal-Anal Impact Questionnaire (CRAIQ-7). Each item in these scales can be scored from 0 (not at all) to 3 (quite a bit), and the number need to be multiplied by 100 and then divided by 3. All these three scales are scored from 0 (least impact) to 100 (greatest impact). A lower score means there is a lesser effect on quality of life. PISQ-12 is commonly used to evaluate sexual function and feelings among patients over the past 6 months (28). It consists of three domains: behavioral/emotive, physical, and partner-related. Each question is rated on a scale that includes response options: 0 (always), 1 (usually), 2 (sometimes), 3 (seldom), or 4 (never). Higher scores indicate better sexual function.

### Statistical Analysis

All statistical analyses were performed by SPSS software version 25.0 (IBM Corporation; United States). General characteristics of participants were analyzed by Student's *t*-test or Pearson's χ*2* test. The prevalence of pelvic floor dysfunction was statistically analyzed with Chi-square test. Mann-Whitney U test was used to compare the pelvic floor muscle strength and the scores of GAD-7, PHQ-9, PFIQ-7 and PISQ-12 between psychological intervention group and control group. The variation of GAD-7 and PHQ-9 scores detected before hysterectomy and 6 months follow-up was determined by Wilcoxon signed-rank test. A threshold of *p* < 0.05 was considered as statistically significance.

## Results

### General Characteristics of Participants

A total of 99 patients underwent hysterectomy were enrolled in this study. Patients were randomly divided into psychological intervention group (*n* = 50) and control group (*n* = 49). The general characteristics of participants in the two groups were described in Table 1. There were no significant differences in patient's age, body mass index (BMI), marital status, educational level, occupation, economic conditions, menstrual status, or surgical method.

**Table 1:** General characteristics of intervention group and control group.

	**Intervention group (*n* = 50)**	**Control group (*n* = 49)**	***p*-value**
Age, M ± SD	50.72 ± 6.20	50.39 ± 7.21	0.783
BMI, M ± SD	23.15 ± 3.27	23.50 ± 2.99	0.414
**Marital status**, ***n*** **(%)**			
Married	48 (96.00%)	44 (89.80%)	0.473
Divorced	1 (2.00%)	3 (6.12%)	
Widowed	1 (2.00%)	2 (4.08%)	
**Educational level**, ***n*** **(%)**			
High school or lower	38 (76.00%)	37 (75.51%)	0.955
University degree or higher	12 (24.00%)	12 (24.49%)	
**Occupation**			
Yes	20 (40.00%)	16 (32.65%)	0.447
No	30 (60.00%)	33 (67.34%)	
**Economic conditions**			
<5,000 CNY /m	15 (30.00%)	19 (38.78%)	0.618
5,000–10,000 CNY/m	19 (38.00%)	15 (30.61%)	
>10,000 CNY /m	16 (32.00%)	15 (30.61%)	
**Menstrual status**			
Latency period	31 (62.00%)	26 (53.06%)	0.368
Menopause	19 (38.00%)	23 (46.94%)	
**Surgical method**			
Laparoscopy	38 (76.00%)	31 (63.27%)	0.168
Laparotomy	12 (24.00%)	18 (36.73%)	

*M ± SD, mean ± standard deviation; BMI, body mass index; CNY, Chinese Yuan*.

### Effectiveness of Psychological Intervention

The evaluative outcomes of patient's psychological status at baseline (T0) and 6-month follow-up (T1) were shown in Table 2. Before hysterectomy, the GAD-7 scores of the patients in intervention group and control group were 5.08 ± 4.97 and 4.27 ± 4.89, without statistically significant difference (*p* = 0.225). After 6 months, the GAD-7 score for intervention group was significantly lower than control group (2.04 ± 1.75 vs. 4.27 ± 3.27, *p* < 0.001). Besides, through 6-month intervention, there was a trend of declination of GAD-7 scores in psychological intervention group (*p* < 0.001), while no significant change was observed in control group (*p* = 0.631). For PHQ-9 scores, the scores in intervention group were significantly lower than that in control group at 6-month follow-up (*p* < 0.001), but no difference was detected before hysterectomy (*p* = 0.148). In addition, after 6-month intervention, the PHQ-9 score was significantly decreased in the intervention group (5.80 ± 4.58 vs. 3.82 ± 2.98, *p* = 0.018). Conversely, patient's PHQ-9 scores in control group were increased (4.69 ± 4.98 vs. 6.78 ± 3.90, *p* = 0.035).

**Table 2:** GAD-7 and PHQ-9 scores of intervention group and control group at baseline (T0) and 6-month follow-up (T1).

	***n***	**GAD-7 score**		***p*-value^a^**	**PHQ-9 score**		***p*-value^a^**
		**T0**	**T1**		**T0**	**T1**	
Intervention group	50	5.08 ± 4.97	2.04 ± 1.75	**<0.001**	5.80 ± 4.58	3.82 ± 2.98	**0.018**
Control group	49	4.27 ± 4.89	4.27 ± 3.27	0.631	4.69 ± 4.98	6.78 ± 3.90	**0.035**
*p*-value^b^		0.225	**<0.001**		0.148	**<0.001**	

a *Mann-Whitney U test*;

b *Wilcoxon signed-rank test. Significant p values are shown in bold type*.

### Pelvic Floor Function at 6-Month Follow-Up

**Figure 2:**
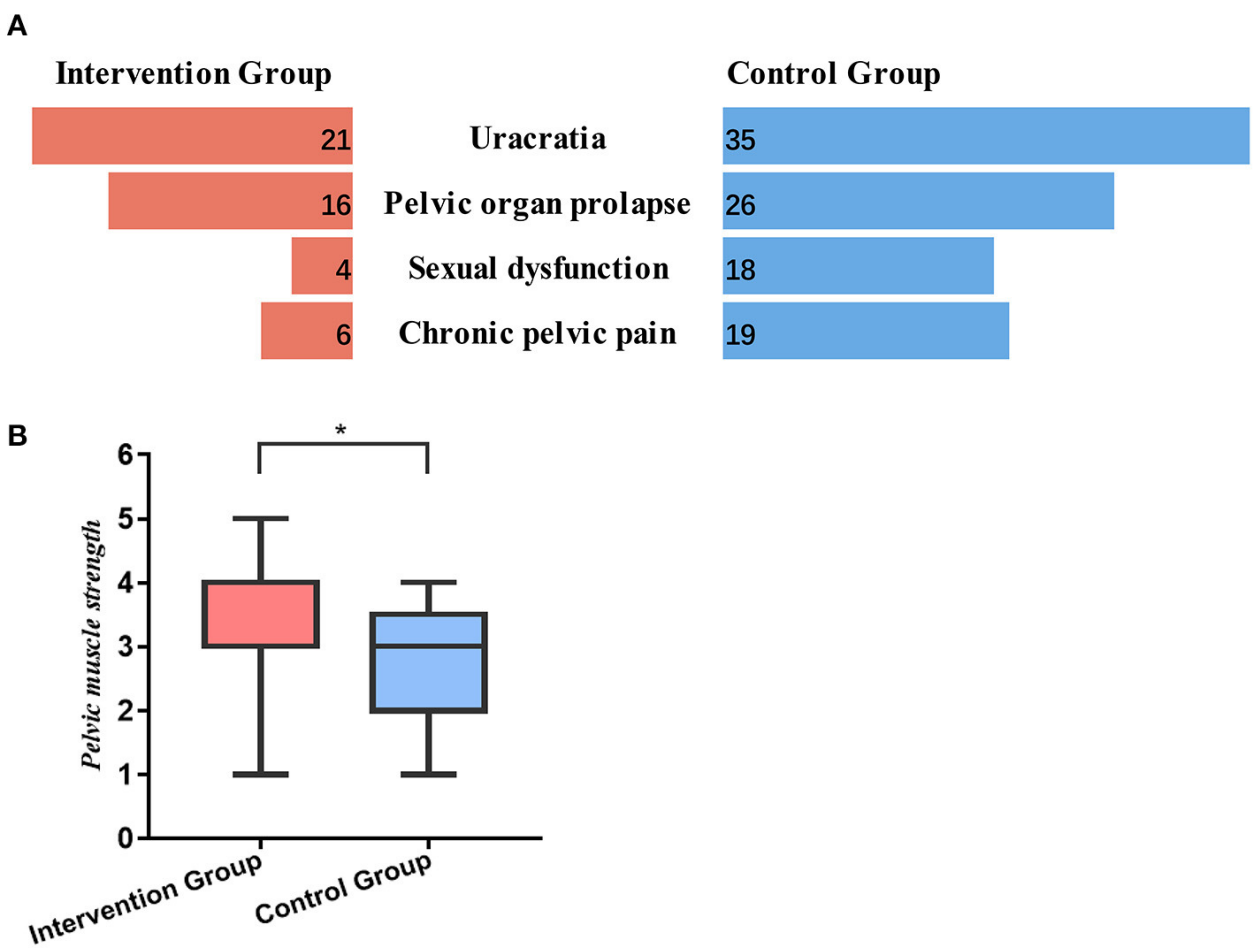
The pelvic floor function of patients at 6-month follow-up. **(A)** The incidence of uracratia, pelvic organ prolapse, sexual dysfunction and chronic pelvic pain in intervention group and control group. **(B)** The pelvic floor muscle strength in intervention group and control group. *, *p* < 0.05.

After 6 months, the patients in two groups presented varying degrees of pelvic floor dysfunction. Therein, the incidence of uracratia, pelvic organ prolapse, sexual dysfunction and chronic pelvic pain for intervention group was 42% (21/50), 32% (16/50), 8% (4/50) and 12% (6/50), respectively. For control group, the rate of these complications was higher, which was 71.43% (35/49), 53.06% (26/49), 36.73% (18/49), and 38.78% (19/49). The differences between two groups were statistically significant (*p* = 0.003, *p* = 0.027, *p* = 0.001, *p* = 0.002). Details were shown in Figure 2A.

We also detected the pelvic floor muscle strength of patients at 6-month following surgery. According to the Figure 2B, the pelvic floor muscle strength in intervention group was significantly stronger than that in control group (3.46 ± 0.994 vs. 2.78 ± 0.872, *p* = 0.001). For control group, nearly half of patient's pelvic floor muscle strength was less than grade 3 (42.85%), while only 18% of patients in intervention group presented with low strength.

### Quality of Life at 6-Month Follow-Up

**Figure 3:**
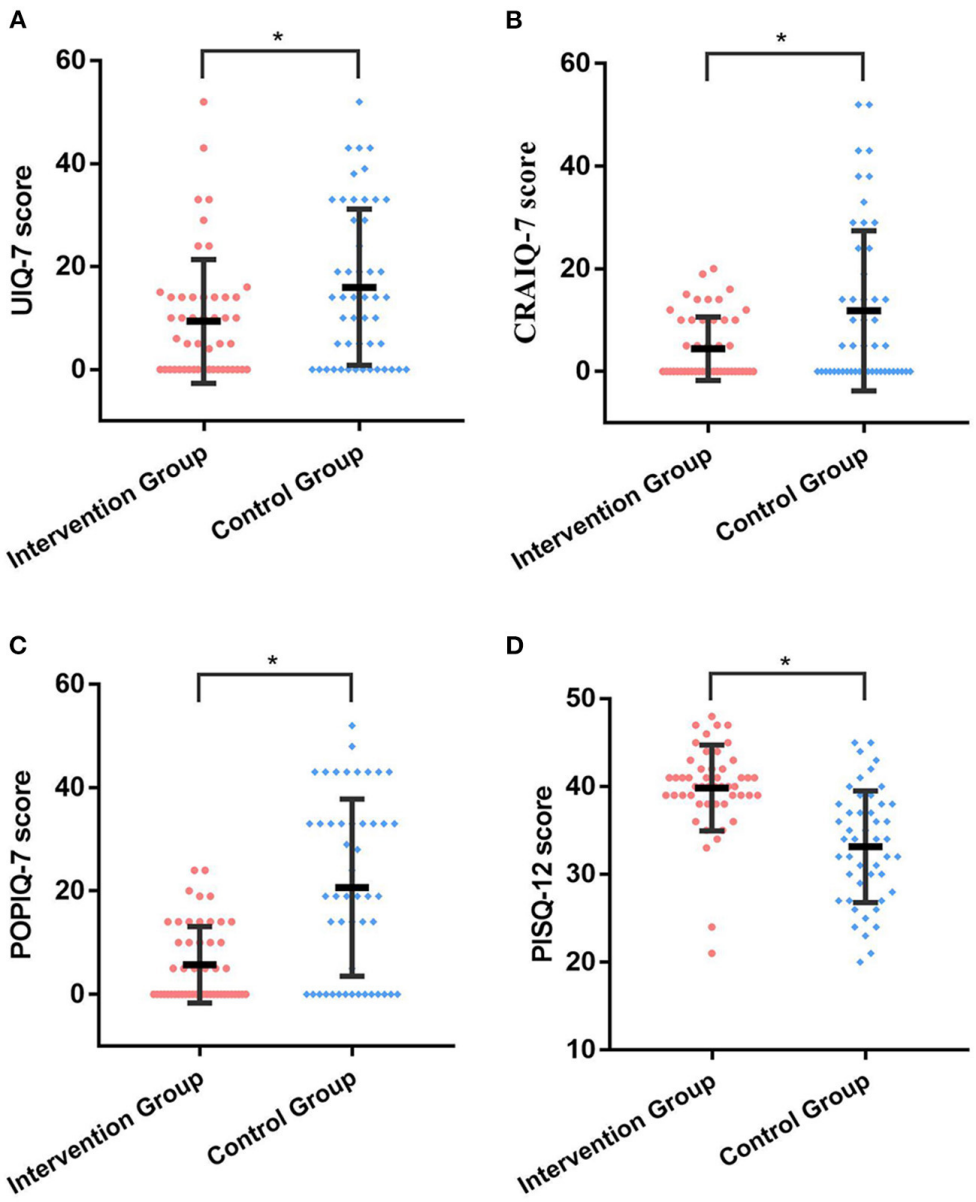
The quality of life for patients at 6-month follow-up. **(A)** The postoperative UIQ-7 scores in intervention group and control group. **(B)** The postoperative CRAIQ-7 scores in intervention group and control group. **(C)** The postoperative POPIQ-7 scores in intervention group and control group. **(D)** The postoperative PISQ-12 scores in intervention group and control group. *, *p* < 0.05.

In order to detect the quality of patients after hysterectomy, we used questionnaires PFIQ-7 and PISQ-12 to evaluate their bladder, bowel, vaginal and sexual function. As shown in Figure 3, postoperative UIQ-7, CRAIQ-7 and POPIQ-7 scores were significantly lower in intervention group than that in control group, and the differences were statistically significant (*p* = 0.025, *p* = 0.04, and *p* < 0.001 respectively). With regard to the sexual function, PISQ-12 score for intervention group was significantly higher than control group (39.84 ± 4.89 vs. 33.14 ± 6.37, *p* < 0.001).

## Discussion

The uterus is an organ with its own special meaning, which performs the important physiological functions of pregnancy and childbirth. It plays an important role in maintaining the confidence and attractiveness of women. Someone feels that loss of uterus means loss of femininity (29, 30). Moreover, hysterectomy fundamentally affects the anatomical structures of pelvic floor, which is considered as a proven risk factor for POP (31, 32). Hence, having a hysterectomy would strongly impact a woman's life, especially her psychological status. In the present study, we intended to explore the efficacy of psychological intervention on the pelvic floor function and psychological outcomes of patients with hysterectomy.

As the advance of surgical techniques, laparoscopic surgeries like robotic single site surgery are feasible and safe for gynecological procedures, thus may improve the quality of life and psychological status of patients (33, 34). However, psychological support is still essential. In our study, six-month psychological care could significantly reduce the anxiety scores and depression scores of patients in psychological intervention group. While for those in control group, the anxiety scores showed no difference between pre-operation and 6-month follow-up. The depression scores of control group were actually increased after 6 months. These results were consistent with the results of previous studies. Lee et al. (35) designed a population-based retrospective cohort study to investigate the risk of postoperative psychiatric disorders in women with hysterectomy. They found 1,381 (14.4%) women experienced psychiatric disorders and 374 (3.9%) experienced mood disorders after hysterectomy. Hence, in addition to physical injuries, patients with hysterectomies may also experience psychological problems (36). Those may be triggered by the negative perceptions about femininity, body image, activity levels, as well as loss of child-bearing capacity (37, 38). Therefore, women who underwent hysterectomies are in great need of psychological care. A study performed by Erdogan et al. (39) confirmed that psychological care had positive effects on depressive symptoms and anxiety of women who underwent hysterectomy. Postoperative nursing should not only focus on the physical issues of patients, but also need to pay attention to their psychological status. As a consequence, appropriate psychological care is necessary for women after hysterectomy, which should become part of routine nursing care.

Hysterectomy fundamentally affects the patient's anatomical structures of pelvic floor, including bowel, bladder, and nerve supply to the area (29). As a result, different degrees of pelvic floor dysfunction were observed in some of patients after hysterectomy. In the present study, compared with control group, patients in psychological group had lower incidence of complications, higher degree of pelvic floor muscle strength and better quality of life. Thus, we believed that psychological intervention could facilitate patient's postoperative recovery in pelvic floor function. Although there have been few reports about the efficacy of psychological intervention on pelvic floor function after hysterectomy, previous studies in other fields had highlighted the availability of psychological screening and intervention in disease treatment (40). A recent meta-analysis of 14 randomized controlled trials, including 1,196 patients, suggested that psychological therapies might have beneficial effects on depression scores and quality of life in patients with inflammatory bowel disease (41). For cancer patients, several clinical trials also demonstrated that psychological intervention could positively affect patient's quality of life and immune response (42–45). Martin et al. (46) evaluated 44 patients with severe pelvic ring injuries and found a strong correlation between pelvic function and depression/anxiety symptoms. Those presented more severe symptoms of depression and anxiety would have lower functional outcomes. Anxiety and depression may contribute to the reduction in patient's quality of life after surgery. Professional psychological intervention would reduce patient's psychological distress and help them cope with life stressors. Besides, psychological intervention could benefit patients in different ways, such as improving their knowledge regarding the postoperative complications, providing professional advices about rehabilitation training, and reducing their psychological distress and concerns (20, 47, 48). Therefore, the combination of disease-directed therapy with psychotherapy should be recommended, which may confer additional and positive functional outcomes.

## Conclusion

Psychosocial stress of patients after surgery and its treatment is often overlooked by physicians. However, it would severely impact patient's quality of life. This study revealed that psychological intervention for patients with hysterectomy could effectively improve the psychological condition, which in advantage of pelvic floor function. These evidence base supported the necessity of psychological interventions in postoperative follow-up care. Therefore, we recommend that psychological intervention should become part of routine nursing care.

## Data Availability Statement

The original contributions presented in the study are included in the article/Supplementary Material, further inquiries can be directed to the corresponding author.

## Ethics Statement

The studies involving human participants were reviewed and approved by Ethics Committee of Guangdong Provincial People's Hospital. The patients/participants provided their written informed consent to participate in this study.

## Author Contributions

Study design and project development was performed by XZ. Data collection and analysis was performed by XZ, MX, XH, SZ, and YC. The first draft of the manuscript was written by XH and MX. All authors contributed to the study conception and design, commented on previous versions of the manuscript, and read and approved the final manuscript.

## Funding

This research was funded by Medical Scientific Research Foundation of Guangdong Province, Grant Number B2020189.

## Conflict of Interest

The authors declare that the research was conducted in the absence of any commercial or financial relationships that could be construed as a potential conflict of interest.

## Publisher's Note

All claims expressed in this article are solely those of the authors and do not necessarily represent those of their affiliated organizations, or those of the publisher, the editors and the reviewers. Any product that may be evaluated in this article, or claim that may be made by its manufacturer, is not guaranteed or endorsed by the publisher.

## Abbreviations

GAD-7: Generalized Anxiety Disorder scale
PHQ-9: Patient Health Questionnaire-9
PFIQ-7: Pelvic Floor Impact Questionnaire
PISQ-12: Pelvic Organ Prolapse/Urinary Incontinence Sexual Questionnaire
UIQ-7: Urinary Incontinence Impact Questionnaire
POPIQ-7: Pelvic Organ Prolapse Impact Questionnaire
CRAIQ-7: Colorectal-Anal Impact Questionnaire
POP: Pelvic organ prolapse
QOL: Quality of life
POP-Q: Pelvic Organ Prolapse Quantitation
BMI: Body mass index.
